# Microbial Diversity and Cyanobacterial Production in Dziani Dzaha Crater Lake, a Unique Tropical Thalassohaline Environment

**DOI:** 10.1371/journal.pone.0168879

**Published:** 2017-01-03

**Authors:** Christophe Leboulanger, Hélène Agogué, Cécile Bernard, Marc Bouvy, Claire Carré, Maria Cellamare, Charlotte Duval, Eric Fouilland, Patrice Got, Laurent Intertaglia, Céline Lavergne, Emilie Le Floc’h, Cécile Roques, Gérard Sarazin

**Affiliations:** 1 UMR MARBEC, Institut de Recherche pour le Développement, Sète-Montpellier, France; 2 UMR LIENSs, Centre National de la Recherche Scientifique, La Rochelle, France; 3 UMR MCAM, Muséum National d’Histoire Naturelle, Paris, France; 4 UMR MARBEC, Centre National de la Recherche Scientifique, Sète-Montpellier, France; 5 Observatoire Océanologique de Banyuls-sur-Mer, Université Pierre et Marie Curie, Banyuls-sur-Mer, France; 6 UMR7154 Institut de Physique du Globe de Paris, Université Paris Diderot, Paris, France; INRA, FRANCE

## Abstract

This study describes, for the first time, the water chemistry and microbial diversity in Dziani Dzaha, a tropical crater lake located on Mayotte Island (Comoros archipelago, Western Indian Ocean). The lake water had a high level of dissolved matter and high alkalinity (10.6–14.5 g L^-1^ eq. CO_3_^2-^_,_ i.e. 160–220 mM compare to around 2–2.5 in seawater), with salinity up to 52 psu, 1.5 higher than seawater. Hierarchical clustering discriminated Dziani Dzaha water from other alkaline, saline lakes, highlighting its thalassohaline nature. The phytoplankton biomass was very high, with a total chlorophyll *a* concentration of 524 to 875 μg chl *a* L^-1^ depending on the survey, homogeneously distributed from surface to bottom (4 m). Throughout the whole water column the photosynthetic biomass was dominated (>97% of total biovolume) by the filamentous cyanobacteria *Arthrospira* sp. with a straight morphotype. *In situ* daily photosynthetic oxygen production ranged from 17.3 to 22.2 g O_2_ m^-2^ d^-1^, consistent with experimental production / irradiance measurements and modeling. Heterotrophic bacterioplankton was extremely abundant, with cell densities up to 1.5 10^8^ cells mL^-1^ in the whole water column. Isolation and culture of 59 Eubacteria strains revealed the prevalence of alkaliphilic and halophilic organisms together with taxa unknown to date, based on 16S rRNA gene analysis. A single cloning-sequencing approach using archaeal 16S rDNA gene primers unveiled the presence of diverse extremophilic Euryarchaeota. The water chemistry of Dziani Dzaha Lake supports the hypothesis that it was derived from seawater and strongly modified by geological conditions and microbial activities that increased the alkalinity. Dziani Dzaha has a unique consortium of cyanobacteria, phytoplankton, heterotrophic Eubacteria and Archaea, with very few unicellular protozoa, that will deserve further deep analysis to unravel its uncommon diversity. A single taxon, belonging to the genus *Arthrospira*, was found responsible for almost all photosynthetic primary production.

## Introduction

Saline inland waters often provide model ecosystems for studying microbial ecology, addressing numerous fundamental questions ranging from microbial diversity to the limits of life in extreme environments [1, 2]. Most saline lakes are endorheic inland water bodies and, as the dissolved mineral composition originates from the evaporation of continental water, are classified as athalassohaline environments [3], from the ancient Greek Θάλασσα (ocean) and άλας (salt), with the α-privative prefix a- (not). These soda lakes are typically alkaline (pH between 9 and 11) and brackish to hypersaline. Such environments are found throughout the world, for example, in the Africa Rift Valley, in Central Asia, in the North American Desert, on the Andean Plateau and in Southern Australia [4]. However, few studies have been carried out into continental thalassohaline systems, where water is allegedly of marine origin and which are mainly found in coastal lagoons, salterns and permanently landlocked systems with hardly any exchange with the sea.

Saline and soda lakes have been widely studied by environmental microbiologists, as they are hosting microbial communities characterized by extreme alkaliphilic and halophilic phenotypes [5]. These environments appear to be much more selective than other aquatic systems, and the members of the eubacterial and archaeal communities found in soda and alkaline lakes are representative of the typical phylogenetic groups of organisms [4] that thrive in extreme environments. Lanzén et al. [1] described the heterotrophic microbial diversity in five soda lakes in the Rift Valley and showed that diversity was positively correlated with pH and salinity. Primary production in such systems is based mainly on phytoplankton photosynthesis, often dominated by cyanobacteria rather than eukaryotic primary producers [6–10]. High photosynthetic rates are sustained by high dissolved inorganic carbon concentrations, whereas the scarcity or absence of zooplankton grazers reduces the top-down control of phytoplankton biomass [11]. On the other hand, marine salterns are typically host to eukaryotic microalgae such as *Dunaliella salina*, with larger, herbivorous metazoan organisms such as brine shrimps *Artemia* spp.

In freshwaters, very high microbial densities and metabolic rates are usually transient and linked to bloom events, occasionally related to nutrient inputs [12, 13], whereas saline and alkaline lakes have permanently high biomass and production rates [11, 14, 15]. Saline and alkaline lakes are, therefore, considered to be among the most productive aquatic systems on Earth [16]. For example, the saline lakes in the Rift Valley harbor high biomasses of cyanobacteria belonging to the genus *Arthrospira*, with a transient dominance of diatoms or chlorophytes (e.g. *Picocystis salinarum*) as well as sharp changes in chlorophyll biomass over the years [9, 10, 17, 18].

Dziani Dzaha Lake (Mayotte, Western Indian Ocean) has permanent green salty water, with occasional gas bubbling, stromatolites on the lake shore, a complete absence of large organisms and is close (0.1 km) to the Indian Ocean, making this lake a very interesting environment for aquatic microbial ecology studies. A chemical analysis was undertaken to determine whether the origin of the lake water was athalassohaline or thalassohaline and the results were compared with those from other inland saline aquatic systems. The microbial diversity of the autotrophic and heterotrophic communities of Dziani Dzaha (*in situ* and on isolated strains) was assessed and compared to the known microbial diversity in saline and alkaline lakes. The relative biomass and cell density of each component of the microbial community were estimated and compared with results from other hypertrophic aquatic systems. Finally, the associated metabolism (photosynthetic oxygen production) and community metabolic potential (use of combined carbon sources) were evaluated.

## Materials and Methods

### Study site

Field permit was granted by: Conservatoire du Littoral et des Rivages Lacustres, Antenne Océan Indien, since Dziani Dzaha is currently a protected water body with free public access but restricted activities, under the control of the French agency for littoral ecosystems conservation (http://www.conservatoire-du-littoral.fr/).

**Fig 1:**
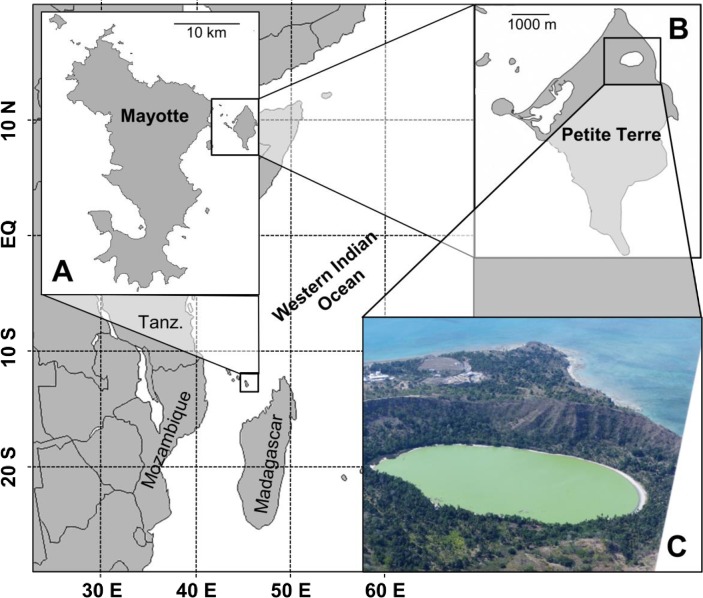
Situation map of study site. (A) Mayotte Archipelago in the Western Indian Ocean, and (B) location of Dziani Dzaha in Petite Terre. (C) An aerial view from an ultra light plane is given, facing North. Picture took in October, 2014, in dry season at low water level. West to East distance between shorelines is 650 m.

Mayotte is an island complex in the Comoros Archipelago in the Northern Mozambique Channel, with two main islands, Grande Terre and Petite Terre, where the study site is located (Fig 1). The island formation probably results from an eruptive event that occurred during the Late Pleistocene / Early Miocene era [19]. The most recent volcanic ash deposits in cores from the surrounding barrier reef were dated from the Holocene, between 7.5 [20] and 4 kyr BP [21], which probably gives the maximum age for Dziani Dzaha lake formation. The lake area is around 25 ha and his altitude (lake surface) close to the mean sea level (the Mozambique Channel shoreline is 230 m to the East), depending on rainfall driven variations. The lake waters have always been dark green so far as local inhabitants remember.

Two complete surveys were conducted in October 2010 and 2011, at the end of the dry season when the lake level was minimal. Previously, two single samples from surface were collected in June 2007 at the end of rainy season (chl *a*), and April 2009 during the rainy season (chl *a*, phytoplankton abundances and biomasses, and dissolved elements) and included in the data set for reference purposes. All measurements and samples were taken from a single station (named CLB for further reference in ongoing studies, position: 12°46’15.6” S; 45°17’19.2” E) at a maximum depth of 4.5 m. Water was collected using a horizontal 1.2 L Niskin bottle for optimum vertical resolution accuracy, and processed on return to the field laboratory within 2 hours.

### *In situ* measurements

Vertical profiles to measure pH, dissolved O_2_, temperature and conductivity were taken using either a MPP350 probe connected to a Multi 350i data logger (WTW GmbH) or a YSI 600XLM probe (YSI). A miniature MkV/L spherical photosynthetically active radiation (PAR) recorder (AlecElectronics) was placed below the surface (0.3 m depth) to record the diurnal underwater light field, and four temperature loggers HOBO Pro V2 (Onset) were positioned every meter in a vertical line. The attenuation coefficient K_d_ was determined from single profiles of discrete PAR measurements taken using a LI-192 underwater quantum sensor connected to a LI-1400 data logger (Li-Cor). Continuous PAR measurements and single vertical light attenuation estimates were used to model the light field in the top meter of the lake, depending on the time of day. Because of field constraints, the incident surface PAR was not measured continuously, and PAR_0.3m_ measurements (I_(0.3,t)_) were used in a modified Beer-Lambert equation to calculate the light field I_(z,t)_ at depth z and time t.

The salinity was calculated using conductivity measured in the vertical profiles. Dissolved oxygen sensors (optode technology, PONSEL Digisens) were suspended in the upper water column at depths of 15 cm and 50 cm, and dissolved oxygen saturation levels were recorded every 5 min. Measurements were converted to mg O_2_ L^-1^ as described in [22], correcting for temperature and salinity. The modeling approach described in [23] was used to calculate the daily gross primary production (GPP) and net ecosystem production (NEP) using discrete O_2_ saturation and temperature measurements. This method is considered to give a robust estimate of oxygen metabolism, as it takes account of physical diffusion. The dissolved oxygen was supersaturated in the upper layer of the lake most of the time and, therefore, some was lost to the atmosphere.

### Nutrients and dissolved elements

The total alkalinity was determined by titration [24]. The ammonium and soluble reactive phosphorus (SRP) were determined colorimetrically using standard test kits (Merck Spectroquant®) as described by the manufacturer, using an AL800 spectrophotometer (Aqualytics) with a 50 mm path length optical glass cuvette.

The dissolved elements were measured using samples diluted in ultrapure water. Chloride was titrated using a standard AgNO_3_ solution (0.5 M), after acidification with analytical grade HNO_3_ (to prevent Ag_2_CO_3_ precipitation). Nitrate and sulfate were measured to ±3% using 20-fold diluted samples by ionic chromatography (Dionex system). Dissolved sodium, potassium, calcium and magnesium were analyzed to ±5% by flame absorption atomic spectrophotometry (ThermoOptek Solaar 6000® FAAS). Dissolved organic carbon (DOC) was measured to ±10% using a TOC analyzer (ThermoFlash 2000®) after acidification with 1% HCl and 20-fold dilution.

The mean Na^+^, Cl^-^, K^+^, Ca^2+^, Mg^2+^, SO4^2^ concentrations and mean alkalinity expressed in mg/L in Dziani Dzaha were compared against data for 34 inland alkaline and/or saline systems as well as standard seawater composition (n = 50, data compiled from [3, 4, 25–29]). Hierarchical clustering using water composition values [30, 31] was performed using PAST 2.13 freeware [32] and Ward’s minimal variance method.

### Abundance and vertical distribution of microorganisms

The vertical distribution of heterotrophic and photosynthetic microorganisms was assessed by cellular enumeration using flow cytometry (unicellular including heterotrophic bacteria and photosynthetic microorganisms), chlorophyll *a* quantification (photosynthetic microorganisms) and microscopic examination (unicellular and filamentous photosynthetic microorganisms).

#### Enumeration of heterotrophic bacterioplankton

Discrete samples (1.4 mL) from various depths were preserved using 0.2 μm filtered formaldehyde solution (4% final concentration) and stored in liquid nitrogen until analysis. The bacterial community was analyzed by flow cytometry using SYBR-green I (Molecular Probes) staining according to [33] using a BD FACScalibur flow cytometer (BD Biosciences). The bacterial populations were determined on the basis of the optical characteristics and the cells were enumerated using CellQuest software (BD Biosciences), using the method detailed in [34].

#### Chlorophyll *a*

Samples for determining the total chl *a* (3–5 mL) were filtered onto 13 mm diameter GF/F filters (Whatman). The contribution of smaller phytoplankton to the total biomass was measured by filtering the larger cells and organisms onto 47 mm diameter polycarbonate membranes with 3 μm porosity (ipPORE). The filters were soaked with 3 mL absolute ethanol, then the cells were sonicated for 30” in an ice bath and extracted overnight at 4°C in the dark. The extracts were filtered again and the absorbance spectrum was measured in the range 400–800 nm using an AL800 spectrophotometer. The chlorophyll *a* content was calculated using the equation formulated in [35]:

$$\left[chl\;a\right]=11,904\;*\left(A_{665}\text{-}A_{750}\right)$$

where [chl *a*] is the concentration of chlorophyll *a* in the ethanol extract in μg mL^-1^ and A_665_ and A_750_ are the absorbance readings at 665 nm and 750 nm, respectively, for a 1 cm path optical glass cuvette.

#### Phytoplankton identification and enumeration

Two sets of samples were prepared for phytoplankton analysis. The first set was fixed immediately in Lugol’s iodine solution and the second set was preserved in neutralized formaldehyde (2% final concentration). Counting was performed using standardized Utermöhl method [36, 37] using an inverted microscope (IX70, Olympus) coupled with a camera (MoticamPro, Motic). Morphological identification was carried out according to reference nomenclatures [38, 39] before further identification by molecular sequencing. Measurements of each quantitative parameter (length, width) were performed on at least 30 individuals of the dominant taxa using an optical microscope (Imager A2 AX10, Zeiss) coupled to an image acquisition system (XCD-U100CR camera, Sony). Biovolumes were calculated for each taxon as described in [40].

#### Diversity and phylogeny of microbial taxa

The microbial taxa were isolated and cultured to select eubacterial strains, both photosynthetic (cyanobacteria) and heterotrophic, using a single water sample collected in October 2011 at a depth of 0.5 m in the middle of the oxygenated layer. Molecular fingerprinting was then used to identify the cultivable heterotrophic Eubacteria strains. Cyanobacteria identification was performed using 16S rRNA gene sequencing. Planktonic Archaea were identified using a cloning-sequencing approach, from the same single water sample, without pre-culturing.

#### Isolation of cyanobacteria

Water samples were inoculated on Z8 medium [41] supplemented with salt (NaCl 20 g L^-1^), in liquid and solid medium (7 g L^-1^ of agar). Cyanobacteria strains were isolated by repeated transfers of trichomes onto solid media (at least 5 steps) under a CK2 inverted microscope (Olympus), and two strains were selected for the present analysis. The cultures were incubated at 27°C, with a 16h light: 8h dark cycle under constant irradiance of 50 μmol photons m^-^^2^ s^-1^ during the light period. Filamentous isolates were obtained and kept in solid and liquid Z8 salt medium, and strains were included in the Paris Museum Collection (PMC, http://mcam.mnhn.fr/Collections/Cyanobacteries.htm).

#### Molecular identification of cyanobacteria

DNA extraction from cyanobacteria strains was made using a commercial kit (QuiaGen, Cat n° 69506) after centrifugation of exponentially growing cultures. Selective amplification of the 16S rRNA gene was performed according to [42] using a FTCPLUS/02 (Techne) thermocycler, with a 5 min denaturation step at 95°C followed by 30 amplification cycles (30 s at 94°C, 30 s at 58°C, 1 min at 72°C) and a 5 min final extension at 72°C. Purified PCR products were sequenced by the GENOSCREEN facility (Lille, France), and the 16S rRNA sequences (1310 bp) were obtained (accession numbers KX840360 and KX840361). Sequence alignment was performed using the ClustalW package of MEGA6 software. Neighbor joining, maximum likelihood and parsimony methods were implemented using the MEGA6 (1000 iterations) for phylogenetic affiliation of cyanobacteria strains. Known sequences (n = 25) from public databases were selected within the order Oscillatoriales according to information available on the collection habitat (extreme environments and / or alkaline-saline lakes), together with *Planktothrix* 16S rRNA gene sequences. *Gloeobacter violaceus* PCC 7421 was used as outgroup.

#### Diversity of cultivable heterotrophic eubacteria

For the cultivation of aerobic heterotrophic bacteria, 1mL of a surface water sample (2011 survey) was mixed with 10% glycerol and immediately put in liquid nitrogen. Back at the laboratory, 3 different solid culture media were selected. In order to mimic the chemical parameters of the lake, each medium was adjusted to have a stable pH of 9.5 and salinity of 56 psu. The 3 media used were: a Marine Agar (MA—Difco Laboratories), a Basalt Salt Methylotroph Medium (BSMM) and a modified BG11 mineral medium [43, 44] used for cyanobacteria isolation.

The most representative cultivable strains were identified for each medium after 2 weeks. The colonies were categorized on the basis of their morphological characteristics. To purify the strains, the various morphotypes (colony morphology) were picked for two successive sub-culturing steps, first onto the same medium and the second onto MA. Each isolate was then grown in marine broth (Marine Broth 2216, Difco Laboratories) for 48 h at 25°C while being agitated (100 rpm). Each culture was cryopreserved in 5% dimethylsulfoxide or 35% glycerol, put into a −80°C freezer and added to the MOLA culture collection (Microbial Observatory Laboratoire Arago, http://collection.obs-banyuls.fr/). The purified strains were genotyped by 16S rRNA gene sequencing (S1 Text).

#### Diversity of archaeal communities

Prokaryotic genomic DNA from a surface water sample (2011 survey) was extracted from a 20 mL subsample prefiltred at 3.0 μm, and collected onto a 0.2 μm filter using the Power Water DNA isolation kit (MoBio Laboratories) as described by the manufacturer for maximum yield. The DNA quality was checked by 1% (w/v) agarose gel electrophoresis and quantified using NanoDrop. The archaeal 16S rRNA full length genes were PCR-amplified using the archaeal specific forward primer Arch21F (5′-TCCGGTTGATCCYGCCGG-3′) and the universal reverse primer 1492R (5′-GGTTACCTTGTTACGACTT-3′) [45], followed by cloning-sequencing (S2 Text).

### Photosynthetic and metabolic potential

#### *In vivo* fluorescence analysis

Pulse-amplitude modulated fluorometry (PAM) was used to determine the photosynthetic potential, which was chosen as a proxy of the health status of the phytoplankton community as a function of the depth. The rationale and classification for fluorescence parameters are reviewed in [46]. A Junior-PAM with a white LED (Walz) was used as described in [47] to record *in vivo* fluorescence transients. 5 mL dark-adapted water samples were filtered onto 25 mm GF/F for 15’ using a pre-soaked paper filter. The light guide was placed over the filter and fluorescence transients were measured using saturating pulses. The dark-adapted quantum yield Qy was calculated using the equation:

$$Qy=\frac{F_{v}}{F_{max}}\;\text{where}\;F_{v}=F_{max}-F_{0}$$

where F_max_ is the maximum chlorophyll fluorescence of the dark-adapted sample subjected to a 600 ms saturating light pulse (>1400 μmol photons m^-^^2^ s^-1^), and F_0_ is the basal fluorescence of the sample at non-actinic light levels (<3 μmol photons m^-^^2^ s^-1^).

#### Production / Irradiance curves

Photosynthetic oxygen evolution (PSOE) was monitored as a proxy for photosynthetic activity. 30 mL samples were distributed in gas-tight polycarbonate flasks. A 5 mm O_2_ sensor optode (PreSens) was glued inside each flask. The flasks were placed one after the other in a custom-made photosynthetron, one side of which was lit by a 4 W white LED to generate a variable flux density up to 1200 μmol photons m^-2^ s^-1^. The PAR was measured in each flask using a US-SQS/L spherical quantum microprobe (Walz) connected to a LiCor-1400 logger (Li Cor). The dissolved oxygen concentration was measured every twenty minutes over the 4–6 h incubation period, the linearity of the oxygen concentration changes over time was checked, and the photosynthetic oxygen evolution (PSOE) rates were plotted against irradiance using the model of Platt et al. [48]:

$$P(I)=P_{max}\times \left(1-e^{\frac{-\alpha I}{P_{max}}}\right)\times e^{\frac{-\beta I}{P_{max}}}$$

The PSOE parameters (α, β and P_max_) obtained from the incubation experiments were used to model the instantaneous gross oxygen production rates at given times depending on the instantaneous light field and the actual chlorophyll *a* biomass. The rationale is described in [49], and the cumulative instantaneous oxygen production rates were used estimate the daily gross O_2_ production per square meter of lake (GPP):

$$GPP\;(g\;O_{2}\;m^{-2}d^{-1})=\int_{dawn}^{dusk}\int_{z=0}^{z=1.5\;m}\left[chl\;a\right]\times P_{max}\times (1-e^{\frac{-\alpha I_{z,t}}{P_{max}}})\times e^{\frac{-\beta I_{z,t}}{P_{max}}}\times dz\times dt$$

Assuming a constant respiration rate, calculated from samples incubated in the dark, the net oxygen production was obtained by subtracting the daily respiration from the daily gross production.

#### Metabolic potential of heterotrophic bacterioplankton

In order to determine the metabolic potential profile of the microbial communities, 150 μL samples from the oxic (0.5 m depth) and anoxic (2 m depth) layers were distributed on Biolog Ecoplates™ (Biolog Inc.) with 31 different organic substrates. Anoxic water samples were inoculated anaerobically in a N_2_-filled hood, and each well was covered by 25 μL of paraffin oil. Another microplate was incubated in air with oxic water samples, and both microplates were kept in the dark for six days at room temperature (26–28°C). Purple color development (from the tetrazolium violet redox dye) was measured daily using a Biorad 680 microplate reader with a 590 nm bandpass filter (Andover Corp.). Data was normalized as described in [50] and the average well color development (AWCD) was used to evaluate the substrate utilization efficiency. The relative efficiency for the various substrates was determined based on the amount (AWCD value) of substrate that was utilized relative to the total amount of substrates (sum of AWCD values of each plate) used by the microbial communities. The 31 carbon sources were grouped to sum the utilization response to all substrates within 6 chemical classes: amines, amino-acids, carbohydrates, carboxylic acids, polymers, and phenolic compounds. The differences in the results for all the samples were tested using the non-parametric Mann-Whitney U-test. Differences were considered as significant at p<0.05 (Sigma Stat version 3.5). Principal Component Analysis (PCA) was performed using ADE-4 [51] to identify the substrates utilized for each functional class by the microbial communities. Differences were considered as significant when p <0.05 (Sigma Stat version 3.5).

## Results

### A warm, turbid and alkaline “marine” lake

**Fig 2:**
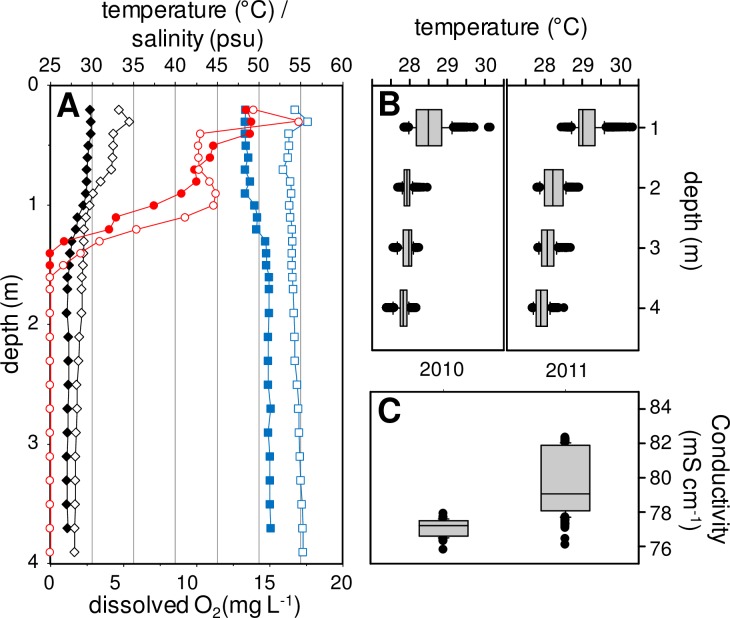
Typical records of vertical profiles. (A) Temperature (°C, black diamonds), salinity (psu, blue squares) and dissolved oxygen concentration (mg L^-1^, red circles) are given for CLB station of Dziani Dzaha Lake in 2010 (closed symbols) and 2011 (open symbols). (B) Box-plot of temperature record at four selected depths during the two surveys (n = 2400). (C) Box-plot of all conductivity records during the two surveys (n = 370 for 2010; n = 252 for 2011).

The temperature was stable within the water column below a depth of 1 m, whereas the surface waters showed daily fluctuations, reaching up to 35°C in the top few centimeters (Fig 2A). On average, at a depth of 1 m the water temperature was 28.5°C in 2010 and 29.1°C in 2011 and 27.8 at a depth of 4 m in 2010 and 27.9°C in 2011 (Fig 2B). In 2010, the mean temperature for the lower water column increased from 27.4°C to 27.9°C (rate of 0.07°C d^-1^), whereas in 2011 it increased from 27.8°C to 28.7°C (rate of 0.08°C d^-1^) during the survey periods.

The lake was very turbid, making Secchi depth estimates unreliable (below 0.05 m). Photosynthetically available radiation measurements were taken by discrete readings using a PAR cosine sensor in 2010 and 2011, and measurements were taken continuously using a spherical probe at a depth of 0.25 m depth during the 2011 survey. The light attenuation coefficient (Kd) was 5.78±0.21 m^-1^ in 2010 and 6.79±0.23 m^-1^ in 2011. The 1% PAR level was, therefore, between 0.7 and 0.8 m below the surface, both years being considered equivalent.

The vertical pH profiles showed little variation throughout the water column, ranging from 9.1 to 9.4 depending on the sampling date (Fig 2A). There was the same pattern for conductivity, with a mean value close to 77.1 mS in 2010 and 79.7 mS in 2011 (n = 82 and 270, respectively, Fig 2C) corresponding to a salinity of around 52.0 psu in 2010 and 52.7 psu in 2011. There was no significant vertical stratification of the water column for pH or conductivity.

It is clear that Dziani Dzaha is characterized by a very high level of dissolved matter and high alkalinity. The high conductivity is in agreement with the high concentrations of Na^+^, Cl^-^ and total alkalinity (Table 1). The elemental ratio compared to seawater is slightly higher for chlorine (40%), more than double for sodium, and more than three times higher for potassium, with an ionic strength 1.5 higher than seawater. The alkalinity is high, with levels comprised between 10.6 and 14.5 g L^-1^ eq. CO_3_^2-^ (around 160–220 mM) throughout the surveys. By comparison with the other saline lakes, Dziani Dzaha is distinguished mainly by high levels of Na^+^, K^+^, Mg^2+^ and Cl^-^, and low levels of Ca^2+^ (S1 Table).

**Table 1:** Concentration of major elements.

Survey	Na^+^	K^+^	Mg^2+^	Ca^2+^	SO_4_^2-^	Cl^-^	TAL
**2009**^**a**^	15290	2025.4	75.9	13.36	185	23567	10632
**2010**	22195	1275.3	108.9	1.48	237.1	27378	14457
**2011**	22149	1314.3	94.8	1.64	278.4	27938	13817

Dissolved anions, cations (mg L^-1^), and total alkalinity TAL (mg L^-1^ eq. CO_3_^2-^) in Dziani Dzaha water.

^a^Sampling at the end of rainy season.

**Fig 3:**
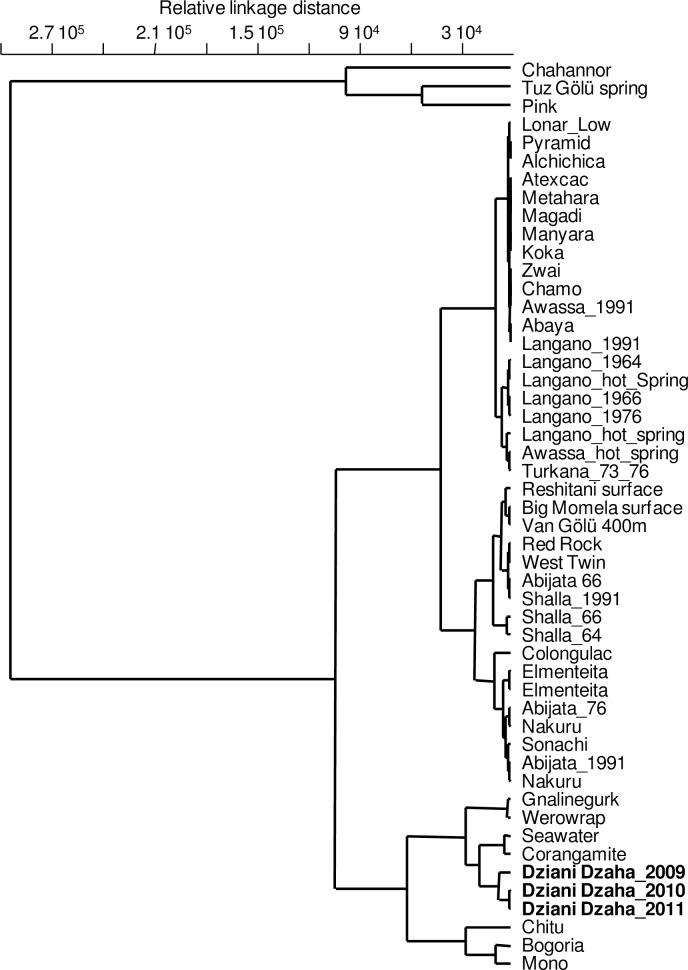
Hierarchical clustering of 50 saline lake waters. Ward’s method clustering based on the records of the six major dissolved elements (Na^+^, K^+^, Mg^2+^, Ca^2+^, SO_4_^2-^, Cl^-^) expressed as mg L^-1^, and total alkalinity expressed as mg L^-1^ eq.CO_3_^2-^.

Hierarchical clustering discriminates Dziani Dzaha waters from most of the other alkaline and/or saline lakes in the comparison (n = 56) and shows that it is closely correlated with standard seawater and the Corangamite Lake (an Australian endorheic salt lake), despite significant differences in alkalinity (Fig 3). Six clusters were distinguished, and PCA showed that most of the differences were due to alkalinity and chloride concentrations (not shown). Two lakes, the Chahannor and Pink Lake, were outliers, with the higher values for chloride, sulfate and sodium concentrations. Cluster I had lakes with high sulfate concentrations and high alkalinity, with moderate sodium and chloride content (S1 Table), and included several Rift Valley lakes and Western Australian water bodies. Cluster II had lakes with lower ionic concentrations, whereas cluster III was relatively uniform Na^+^ and Cl^-^ at median concentrations. Clusters II and III were poorly discriminated by the PCA. Two clusters grouped lakes with waters close to standard seawater: cluster Va with 4 Australian lakes and one Andean lake with low alkalinity, and cluster Vb with high alkalinity and balanced Na^+^ and Cl^-^ concentrations, including Dziani Dzaha.

### A stable, healthy autotrophic community with low species richness

**Fig 4:**
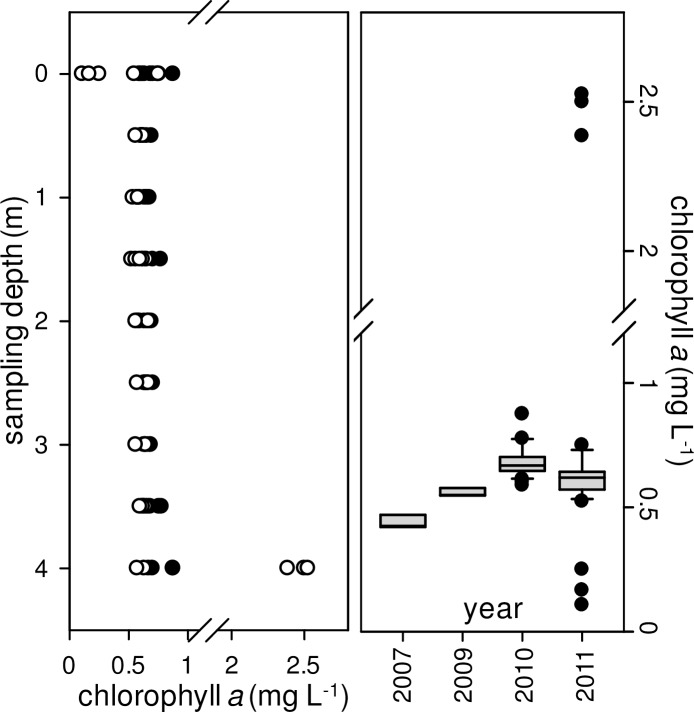
Chlorophyll *a* dynamics in the Dziani Dzaha during 2010 and 2011 surveys. (A) Sum of all chl *a* measures depending on sampling depth for 2010 (closed circles, n = 27) and 2011 (open circles, n = 37) surveys. (B) Box-plot indicating median, 10th and 90th percentiles (whiskers) and outliers (closed circles) for chl *a* concentrations including single samples collected in 2007 (n = 3) and 2009 (n = 3), and 2010 (n = 33) and 2011 (n = 47) surveys.

The total chlorophyll *a* concentration in Dziani Dzaha was extremely high, mainly around 524 μg chl *a* L^-1^ during the 2010 survey and 875 μg chl *a* L^-1^ in 2011 (n = 74), with a uniform vertical distribution (Fig 4A). Without outliers, the mean values were 685±66 μg chl *a* L^-1^ in 2010 and 702 ± 48 μg chl *a* L^-1^ in 2011. These values were not significantly different from those of the single samples previously recorded in 2007 and 2009 (Fig 4B). There was only one exception on Sep. 29^th^, 2011, when the surface values dropped to 175 μg chl *a* L^-1^ with the concentration reaching 2470 μg chl *a* L^-1^ just above the sediment (outliers in Fig 4A and 4B). The contribution of picoplankton cells to total chl *a* (chl *a* < 3 μm) was relatively stable over time and space and accounted for only 6.1%±1.5 (n = 30) of the total pigment content.

The photosynthetic parameters calculated from the fluorescence measurements (n = 71) were similar for both years and there were no significant differences in relative electron transport rate (rETR) curve parameters (rETRmax, α, and β) or quantum yield Qy (one-way ANOVA) over time or sampling depth (data not shown). The average quantum yield during each survey was 0.332±0.036 (n = 44) in 2010 and 0.303 ±0.060 (n = 27) in 2011. The sampling depth (0–4 m) did not affect the apparent healthy state of the planktonic photosynthetic community.

**Fig 5:**
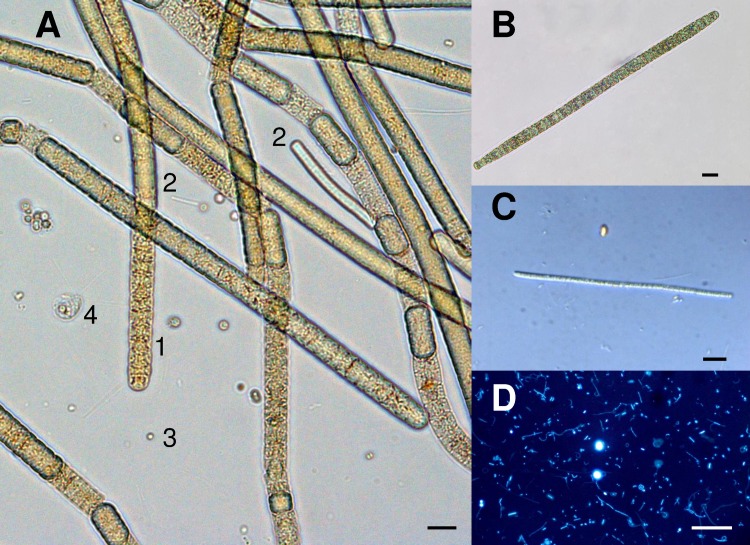
Microscopical photographies of plankton samples of Dziani Dzaha (surface sample, 2011 survey). (A) Total phytoplankton community after Lugol staining with large *Arthrospira* sp. trichomes (1), thin filaments belonging to the Leptolyngbyaceae family (2), unidentified coccoidal cells (3), and large eukaryotic colored flagellate (4). (B) General view of *Arthrospira* sp. (C) General view of thin filaments of cyanobacteria belonging to the Leptolyngbyaceae family. (D) Epifluorescence microscopy picture after SybrGold staining of nucleic acids, highlighting bacterioplankton morphological diversity. Scale bar: 10 μm.

**Fig 6:**
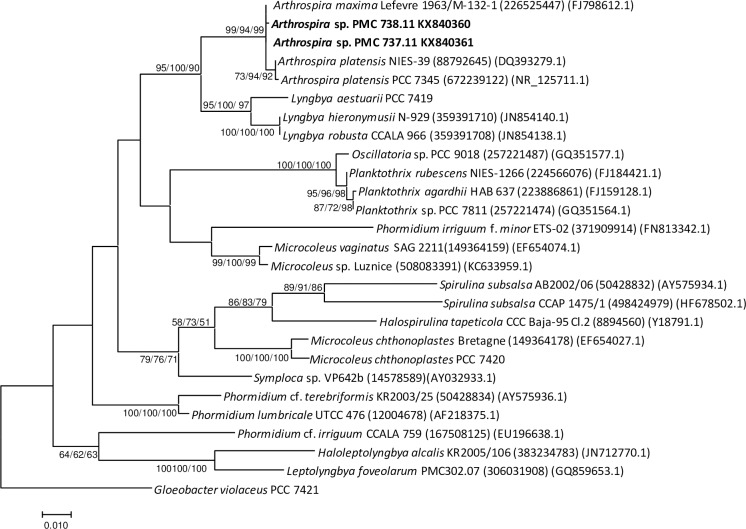
Consensus phylogenetic tree (maximum likelihood) constructed using 16S rRNA gene sequences (1310 bp). Clustering of Dziani Dzaha *Arthrospira* sp. (in bold) with related Oscillatoriales species is provided with accession number and collection codes. *Gloeobacter violaceus* PCC7421 was used as outgroup. Number near nodes indicates bootstrap values >50% for neighbor joining, maximum parsimony and maximum likelihood analyses.

Microscopic examination revealed that a filamentous cyanobacterium corresponding to the morphology of a straight *Arthrospira* (Fig 5A and 5B) dominated, accounting for 99.2 and 97.5% of the total biomass in 2010 and 2011, respectively (Table 2). Classification of this cyanobacterium into the *Arthrospira* genus was based on the following morphological characteristics: trichome cylindrical, not or slightly constricted at the cross-walls, gradually attenuated at the ends, apical cell rounded often with thickened outer cell wall, cells shorter than wide (8.02–9.45 μm wide, 3.4–3.65 μm long), cross-wall visible (under light microscope), motile (gliding, rotation), cell content olive-green with numerous aerotopes. Sheaths were facultative. The identification as *Arthrospira* using the morphological approach was confirmed by 16S rRNA gene sequence analyses on the two cultivated isolates (PMC 737.11 and 738.11). Molecular analyses revealed that this cyanobacterium shared 99% sequence identity with *Arthrospira platensis* and *A*. *maxima* (Fig 6). Less dominant taxa were represented by several morphotypes of thin filamentous cyanobacteria (<3μm) belonging to the family Leptolyngbyaceae (Fig 5A and 5C). Together with these filamentous forms, numerous round cells were visible using microscopy, without any straightforward distinction between picocyanobacteria and eukaryotic picophytoplankton. There were a few large eukaryotes, mainly with motile forms, one smaller (6–8 μm) with two flagella and one larger (12–16 μm) with four flagella. The overall species richness of primary producers appears, therefore, to be low when evaluated using only optical microscopy.

**Table 2:** Relative phytoplankton contribution (%) to abundances (individuals mL^-1^) and total biomass (μg L^-1^) for samples taken during the rainy (2009) and dry seasons (2010 and 2011).

	% abundance			% biomass*		
Survey	2009	2010	2011	2009	2010	2011
*Arthrospira* sp.	12.6	11.6	6.8	98.1	99.2	97.5
Leptolyngbyaceae	23.2	49.3	16.4	1.7	0.55	1.4
Picocyanobacteria + picoeucaryotes	64.2	38.9	74.2	0.3	0.17	0.56
Eukaryotic autotrophic flagellates	-	0.2	2.6	-	0.08	0.54

* Biomass was obtained from biovolume calculations for microscopic observations, and carbon-to-cell conversion for picophytoplankton enumerated by flow cytometry.

### A thin but highly productive well-lit layer

Oxygen concentration was very high at the top of the water column, reaching over 200% of saturation at midday, and decreased sharply to zero at depths below 1.5 to 2 m (Fig 2A). The subsurface diurnal cycles recorded during the 2011 survey showed a clear day/night pattern, but without total nocturnal depletion of the dissolved oxygen. At 31°C and 52 psu, the oxygen saturation corresponded to a concentration of 5.6 mg O_2_ L^-1^ in the top 50 cm layer, and the dissolved oxygen never dropped far below this value even at night time.

The oxygen variation in the lake, measured at depths of 15 cm and 50 cm in 2011, showed a clear pattern of net production during the daytime and net consumption at night (S1 Fig). Linear regression of dark records gave a respiration rate of between -0.93±0.024 mg O_2_ L^-1^ h^-1^ and -0.59±0.012 mg O_2_ L^-1^ h^-1^ depending on the depth and day considered (Table 3), whereas the apparent *in situ* net production during the first three hours of the day varied between 1.19±0.06 mg O_2_ L^-1^ h^-1^ and 3.59±0.2 mg O_2_ L^-1^ h^-1^. Salinity remained almost constant (51.3–52.7 psu) during the two days of dissolved oxygen measurement. The GPP was 22.2 g O_2_ m^-^^2^ d^-1^ for first day and 17.3 g O_2_ m^-^^2^ d^-1^ for the second day, whereas the net ecosystem production NEP was close to equilibrium: 1.97 g O_2_ m^-^^2^ d^-1^ for the first day and -0.90 g O_2_ m^-^^2^ d^-1^ for the second day (Table 3).

**Table 3:** Estimate of daily gross primary production (GPP), respiration (R) and net ecosystem production (NEP) integrated over the upper photic layer of Dziani Dzaha, expressed as g O_2_ m^-2^ d^-1^.

		GPP	R	NEP	P/R
optodes *in situ*	day 1	22.2	-20.3	1.97	1.09
	day 2	17.3	-18.2	-0.90	0.95
PSOE	optimal	19.4	-11.4	8.09	1.70
	upper limit	24.6	-11.4	13.3	2.16
	lower limit	14.4	-11.4	3.0	1.26

Staehr et al. [23] method was used to fit optode *in situ* measurements, whereas Bright and Walsby [49] method was used to model production integral according to laboratory photosynthetic oxygen evolution (PSOE) measurements.

The PAR range applied in the photosynthetron varied from zero to 1100 μmol photon m^-2^ s^-1^, enough for light saturation of *in vitro* photosynthetic activity. The mean values for maximum oxygen production rates (P_max_) ranged from 5.61 to 11.76 mg O_2_ mg chl *a*^-1^ h^-1^ (equal to 175–368 μmol O_2_ mg chl *a*^-1^ h^-1^) during the 2011 survey and the PSOE half-saturation flux density was 99 μmol photons m^-2^ s^-1^. Higher flux densities decreased the PSOE, showing significant photoinhibition (Table 4). The light efficiency α (initial slope of the P/E curve) was 3.31±1.75. Both P_max_ and α values, calculated from the oxygen production measurements, are within the standard range reported for cyanobacterial blooms.

**Table 4:** Parameters of photosynthesis / irradiance experiments.

method	PAM (2010)	PAM (2011)	PSOE (2011)
Pmax^§^	46.7 ± 7.9	40.6 ± 12,4	271 ± 73
α	0.156 ± 0.,040	0.189 ± 0.064	3.31 ± 1.75
β	0.007 ± 0.004	0.009 ± 0.008	0.109 ± 0.104
Ik (μmol photons/m^2^/s)	309 ± 81.9	225 ± 65.3	88 ± 39.6
*n*	53	23	8

§ productivity is expressed as μmol electrons m^-2^ s^-1^ for PAM fluorometry, and as μmol O_2_ mg chl *a*^-1^ h^-1^ for photosynthetic oxygen evolution.

Using α, β and P_max_ derived from PSOE curves, the daily oxygen production was calculated using the method described in [49]. Using the input values listed in Table 4 for PSOE, a constant [chl *a*] of 612 μg L^-1^ over the whole well-lit layer (mean of 2011 values excluding outliers), the averaged light measurements at a depth of 0.3 m (mean of 10 days, n = 720 for daytime periods) and a PAR attenuation coefficient Kd = 6.79 m^-1^, the instantaneous O_2_ gross production was calculated for 10 min intervals at a 10 cm resolution, giving a GPP of 19.4 g O_2_ m^-2^ d^-1^ (Table 3). Assuming a constant mean respiration rate, for the whole period, equal to 0.6 mg O_2_ L^-1^ h^-1^ calculated from incubations in bottles in the dark, the resulting net production was 8 g O_2_ m^-^^2^ d^-1^ (Table 3). The GPP modeling was sensitive mainly to uncertainties in P_max_, Kd, α and β (in descending order). Adjusting the input values by ±½ SD varied the GPP between 14.4 and 22.3 g O_2_ m^-^^2^ d^-1^.

### High abundance, diversity and activity of heterotrophic bacteria and archaea

#### Bacterioplankton abundance

**Fig 7:**
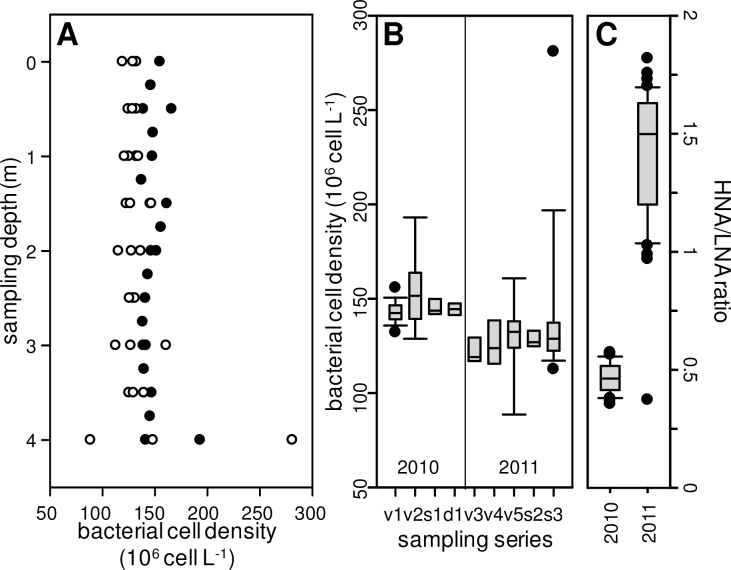
Bacterioplankton cell densities in the Dziani Dzaha. (A) Vertical profiles from discrete samples collected during 2010 (closed symbols) and 2011 (open symbols) surveys. (B) Box-plot indicating median, 10th and 90th percentiles (whiskers) and outliers (closed circles) for bacterioplankton cell densities depending on sampling series: v = vertical profiles; s = surface spatial sampling; d = deep spatial sampling. (C) Box-plot (same attributes) of high- to low-DNA content bacteria ratio for 2010 and 2011 surveys.

The bacterioplankton densities were extremely high for all the samples analyzed and flow cytometry analysis showed that there were four main populations, differing in size and apparent nucleic acid content. Morphology of bacterioplankton appeared also very diverse using epifluorescence microscopy (Fig 5D, for illustrative purposes).The vertical distribution was uniform from surface to bottom, except for a sharp increase in the total bacterial cell density at the bottom of the lake on one occasion (Fig 7A). No significant differences were found in the total bacterial cell counts between days of year, years or sampling depths, with an average density over the whole survey of 1.38 x 10^8^ cells mL^-1^ (SD = 22.4%, n = 77, Fig 7B). However, there was a significant difference (p<0.001, t-test) between the two years in the high-to-low nucleic acid ratio (Fig 7C), with a mean HNA/LNA value of 0.46 in 2010 (n = 35) and 1.41 in 2011 (n = 42).

#### Cultivable bacterial diversity

Fifty-nine strains (Table 5) were isolated on the three media (42 on MA, 14 on BSMM and 3 on BG11). Analysis of the partial 16S rRNA gene sequences found 33 different phylotypes (with at least 1 different base pair). The diversity on BSMM was similar to that on MA except for the MOLA1057 strains which might be a new species in the genus *Bacillus* (S1 Text). The three strains isolated on BG11 were all different from those on the other two media. No phototrophic organisms were isolated.

**Table 5:** List of the 59 Eubacteria strains isolated from Dziani Dzaha.

MOLA strain #	Closest valid relative species in Ez Biocloud database	Ecosystem	16S ID (%)	family
1061**	*Marinobacter lipolyticus*	hypersaline	97.53	*Alteromonadaceae*
1059**	*Chromohalobacter salexigens*	moderate halophile	100	*Halomonadaceae*
1056*	*Halomonas meridiana*	Antarctic saline lake	99.88	*Halomonadaceae*
1104*	*Halomonas mongoliensis*	soda lakes, Mongolia	97.64	*Halomonadaceae*
1060**	*Halomonas johnsoniae*	alkaline, halo-tolerant, infectious	98.8	*Halomonadaceae*
1031	*Halomonas salifodinae*	salt mine, China	99.88	*Halomonadaceae*
1062	*Halomonas stevensii*	n.d.	99.65	*Halomonadaceae*
1029, 1030	*Halomonas venusta*	halophile, potentially infectious	99.77	*Halomonadaceae*
1037	*Aliidiomarina maris*	solar saltern, Korea	99.88	*Idiomarinaceae*
1045	*Aliidiomarina maris*	solar saltern, Korea	98.48	*Idiomarinaceae*
1028	*Aliidiomarina shirensis*	seawater, Taiwan	95.59	*Idiomarinaceae*
1018, 1019	*Aliidiomarina shirensis*	seawater, Taiwan	96.63	*Idiomarinaceae*
1036, 1039, 1040,1042	*Nitrincola lacisaponensis*	Soap Lake, USA	99.22	*Oceanospirillaceae*
1067	*Nitrincola lacisaponensis*	Soap Lake, USA	99.46	*Oceanospirillaceae*
1026, 1055*	*Vibrio metschnikovii*	blood samples	98.75	*Vibrionaceae*
1022, 1027, 1034, 1065	*Alkalimonas collagenimarina*	sediment, deep-sea, Japan	96.99	*Unclassified Gammaproteobacteria*
1068	*Cryomorpha ignava*	polar habitat	89.87	*Cryomorphaceae*
1038, 1063, 1064, 1099*, 1100*	*Cecembia lonarensis*	Lonar Lake, India	95.58	*Cyclobacteriaceae*
1047, 1097	*Salinarimonas ramus*	oil contaminated saline soil, China	96.14	*Bradyrhizobiaceae*
1102*	*Nitratireductor pacificus*	HAP-degrading, sediment, Pacific	98.13	*Phyllobacteriaceae*
1101*	*Porphyrobacter dokdonensis*	seawater, Dokdo Island, Korea	100	*Rhodobacteraceae*
1025	*Porphyrobacter tepidarius*	hot spring, Japan	98.41	*Rhodobacteraceae*
1105*	*Rhodobaca bogoriensis*	Lake Bogoria, Rift Valley	98.94	*Rhodobacteraceae*
1103*	*Rhodobaca bogoriensis*	Lake Bogoria, Rift Valley	99.83	*Rhodobacteraceae*
1066, 1050, 1051, 1098	*Inquilinus limosus*	human sample, pathogen	92.1	*Rhodospirillaceae*
1046	*Amphibacillus jilinensis*	soda lake sediment, China	99.21	*Bacillaceae*
1023, 1024, 1058*	*Bacillus agaradhaerens*	alkaliphilic	100	*Bacillaceae*
1043, 1044, 1048	*Bacillus aurantiacus*	soda lake, Hungary	98.03	*Bacillaceae*
1033	*Bacillus horikoshii*	soil, toxin-producer	98.96	*Bacillaceae*
1020, 1021, 1032,1052*, 1053*, 1054*	*Bacillus pseudofirmus*	soil	99.88	*Bacillaceae*
1057*	*Bacillus saliphilus*	mineral pool, Italy	96.11	*Bacillaceae*
1041	*Bacillus saliphilus*	mineral pool, Italy	100	*Bacillaceae*
1035	*Bacillus zhanjiangensis*	oyster, China	98.62	*Bacillaceae*

The nature of selective media (MA, BSMM*, and BG11**) with number in the MOLA collection and phylogenetic assignment to the eubacteria family level using 16S rRNA gene homology (% homology to the closest relative match) are provided.

The 59 cultivated and isolated strains were affiliated to the classes Gammaproteobacteria (42%), Alphaproteobacteria (19%), Bacilli (29%) and Cytophaga / Flavobacteria (10%). Thirteen different taxonomic families divided into 16 genera were identified. The isolates affiliated to the genera *Aliidiomarina* (5), *Alkalimonas* (4), *Bacillus* (16), *Cecembia* (5), *Halomonas* (6), *Nitrincola* (5), *Rhodobaca* (2) and *Vibrio* (2) had already been found in similar halo-alkaline conditions [52–55]. However, the 16S rRNA gene sequence analysis may have revealed new taxa (Table 5 and S1 Text). Two of the five strains could be considered to be new genera (<93%) within the Rhodospirillaceae and Cryomorphaceae families while seven strains (out of 17) that shared between 96% and 98% of sequence identity could represent new species within the Halomonadaceae (Gammaproteobacteria) and Bradyrhizobiaceae (Alphaproteobateria) families.

#### Archaeal diversity

A total of 78 sequences were retrieved using archaeal-specific 16S rRNA gene primers. Seventeen different operational taxonomic units (OTUs) were defined as sequence groups in which sequences differed by less than 3% (S2 Text). All sequences were related to the Euryarchaeota phylum, most of them belonging to groups thriving in extreme environments: Halobacteria (4 unique sequences, 6 clones), Thermoplasmata (7 unique sequences, 61 clones), and Methanomicrobia (5 unique sequences, 11 clones). Halobacteria are typical of hypersaline habitats, whereas Thermoplasmata are commonly retrieved from hydrothermal vents and hot springs. Seven sequences for 35 clones were in a cohesive unassigned branch. These were related to sequences retrieved from hydrothermal vent archaea (*Methanopyrus kandleri*) and several uncultivated sludge and benthic ecosystems. The 17 sequences described in this study were added to the INSDC databases, DDBJ, EMBL and Genbank, under accession numbers KT715014 to KT715030.

#### Bacterial functional diversity

Color development on Biolog Ecoplates started to develop in less than 24 h after inoculation with Dziani Dzaha water. Substrate conversion and color development increased exponentially over 3 to 4 days and the results at 4 days were chosen for further analysis. The incubation conditions, anaerobic or aerobic, did not have any significant effect on the color development kinetics were observed for a given sample (data not shown). A sample was selected from each depth for each survey: from 0.5 m, incubated under air (OX10 and OX11) and from 4 m incubated under nitrogen + paraffin oil (AN10 and AN11) for 2010 and 2011. Substrate utilizations, grouped by chemical classes of compounds, were found to be different in 2010 and 2011 (Table 6). Except for incubation under aerobic conditions in 2010, carbohydrates were the most used compounds, followed by amino acids and carboxylic acids. The major differences between the two years were in efficiency of polymer use (≈23% in 2010, 9.2 and 13.8% in 2011). Anaerobic conditions favored the metabolism of amines, whereas amino acids were degraded more efficiently under aerobic conditions.

**Table 6:** Percentages of substrate (regrouped by chemical classes) utilization for each sampling condition during the two experiments in 2010 and 2011.

	2010		2011	
depth	0.5 m	4 m	0.5 m	4 m
conditions	aerobic	anaerobic	aerobic	anaerobic
Amines	0.3	8.7	1.1	6.2
Amino-acids	32.0	16.2	26.5	20.3
Carbohydrates	23.1	28.2	47.6	43.1
Carboxylic acids	19.8	16.8	15.2	13.2
Phenolic compounds	1.9	6.4	0.4	3.3
Polymers	23.0	23.7	9.2	13.8
AWCD^a^	0.284	0.567	0.482	0.595

^a^Average well color development (AWCD) values after 4 days of incubation are reported.

**Fig 8:**
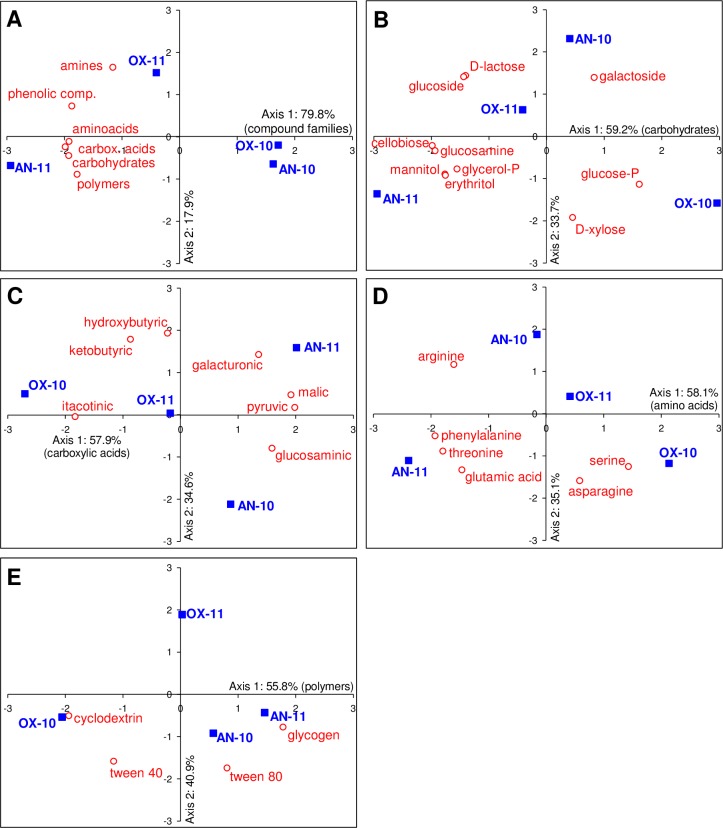
Principal component analysis (PCA) of Biolog ECO plates community metabolic potential. AWCD after 96 h incubation was used as input data. Samples are represented by blue squares, with OX and AN for oxic and anoxic conditions respectively, with sampling year provided. PCA were performed for compound merged (A) in families, or molecules within a chemical group: (B) carbohydrates, (C) carboxylic acids, (D) amino acids, and (E) polymers.

Principal Component Analysis (Fig 8) was used to analyze the differences in the metabolic potentials based on the utilization of different families of compounds. They were very similar for aerobic incubation of samples from 0.5 m deep (OX10) and for anaerobic incubation of samples from 2 m deep (AN10) in 2010 but they were significantly different in 2011 (Fig 8A). In 2011, the community in the oxic sample (OX11) was most efficient at metabolizing amines and phenolic compounds, whereas carbohydrates and polymers were more rapidly degraded in the anoxic sample (AN11). Of the carbohydrates, glucose phosphate and D-xylose were utilized preferentially in the 2010 oxic sample (OX10) although glycerol phosphate, mannitol, glucosamine, and cellobiose were utilized preferentially in the 2011 anoxic sample (AN11) (Fig 8B). Of the carboxylic acids, itaconic acid was utilized most efficiently in the oxic samples, especially in 2010 (OX10), and hydroxybutyric and ketobutyric acids were not utilized (Fig 8C). Of the 6 amino acids, glutamic acid, threonine and phenylalanine were metabolized preferentially in the 2011 anoxic sample (AN11) and asparagine and serine in the 2010 oxic sample (OX10) while the 2011 oxic sample (OX11) showed low amino acid utilization (Fig 8D). Of the polymers, tween 80 and glycogen were the main compounds used in the anoxic samples (AN10 and AN11) while tween 40 was metabolized in the 2010 oxic sample (OX10) but not in the 2011 oxic sample (OX11) which had no affinity for any polymer substrate (Fig 8E).

#### Estimates of organic carbon and nitrogen standing stocks in the water column

Dissolved organic carbon concentrations were relatively homogeneous on the water column, with a mean of 77.5 mg L^-1^ (n = 11) in 2010 and 66,6 mg L^-1^ (n = 25) in 2011, and C:N ratio for dissolved organic matter enriched in carbon relative to particulate matter (Table 7). Total particulate organic carbon and nitrogen were also higher during the 2010 survey than the 2011 survey, with a more balanced C:N ratio close to twelve (Table 7). The contribution of living matter to total suspended solids was estimated by cumulating the biovolumes of phytoplankton enumerated by microscopy to flow cytometry counts of picophytoplankton for autotrophs (Table 2), and cytometry counts for heterotrophic bacterioplankton (Fig 4), with published biovolume-to-carbon and cell-to-carbon conversion factors [56].

**Table 7:** Standing stocks (mean and range) of dissolved and particulate organic carbon (DOC and POC) and dissolved and particulate organic nitrogen (DON and PON) in the pelagic of Dziani Dzaha during 2010 and 2011 surveys.

		2010	2011
DOC	mg/L	77.5 (69.1–85.5)	66.6 (56.4–70.9)
DON	mg/L	4.80 (4.42–5.13)	5.74 (5.42–6.06)
C:N (dissolved)		18.9	15.5
POC	mg/L	82.7 (50.7–126.4)	58.2 (48.3–66.9)
PON	mg/L	7.8 (4.0–11.6)	5.8 (3.0–9.2)
C:N (particulate)		12.4	11.7
POC autotrophs	mg/L	39.3 (31.5–42.8)	34.7 (30.2–39.1)
POC heterotrophs	mg/L	4.5 (4.2–4.8)	4.1 (3.8–4.4)

POC estimation for autotrophs and heterotrophs is based on biovolume-to-carbon and cell-to-carbon estimates [56]. C:N are dimensionless, based on molar conversion.

Organic carbon and nitrogen stocks in dissolved and particulate matter are roughly comparable, and autotrophic biomass accounted specifically for almost 47 and 59% of total POC in 2010 and 2011 respectively, compared to 4.5 and 7% for heterotrophic bacterioplankton for the same surveys.

## Discussion

Dziani Dzaha is a shallow lake, with no vertical stratification during dry season except for dissolved oxygen, and the water buffering characteristics explain the pH stability despite high primary production rates at the surface. Despite the chemical composition classifying it as halo-alkaline (S1 Table), the dissolved element composition in Dziani Dzaha appears to be extremely unusual. Hierarchical clustering is mainly driven by the sodium and chloride concentrations, which are the main factors explaining the similarity with standard seawater (Fig 3). The origin of the Dziani Dzaha water is most probably from the nearby ocean through bedrock seepage and geological fracturation, with subsequent modifications by precipitation inputs, hydrothermal activity, precipitation of dissolved elements and biogeochemical activity, as shown in many alkaline environments where photosynthetic organisms drive high pH conditions [8]. This marine origin is unlike that of most known soda lakes, which are inland water bodies whose salinity is due to dissolved continental minerals [4].

The high turbidity and low light penetration in Dziani Dzaha is typical of hypereutrophic ponds and lagoons [57] where resident phytoplankton and suspended detritic material absorb most of the incident light in the upper water layer. As a consequence, only the top meter of the lake is photosynthetically active. The consequence for the cyanobacterial community is a sharp spatial segregation between active photosynthetic filaments under the surface and photosynthetically quiescent filaments below this layer. Relative photosynthetic activities were high, falling into the same range as rETR values reported for cyanobacterial cultures [47, 58] and blooms [59]. The uniform transient fluorescence characteristics, over time and all sampling depths, strongly indicates that the cyanobacterial populations were always in good health, so far as photosynthetic activity was concerned, and were uniform and stable even in the lowest part of the water column.

The high chlorophyll *a* concentration, coupled with a tropical location with high solar irradiance, resulted in high O_2_ production and allowed the calculation of daily production rates. According to [23], in eutrophic lakes the uncertainties are reduced even when input values such as piston velocity and O_2_ saturation in the water body are approximated. For carbon production, assuming a photosynthetic quotient PQ (molar ratio of O_2_ evolved to CO_2_ fixed) equal to 1 [9], the two days of dissolved oxygen measurements were consistent with a daily carbon fixation of 8.3 g C m^-2^ d^-1^. If it is constant, this activity gives an annual organic carbon fixation of 3.0 10^3^ g C m^-2^ (75 10^6^ g C over an area of 25 ha). This is consistent with the highest reported values for shallow tropical lakes dominated by cyanobacteria, such as the African Rift Valley soda lakes (6.8–10.7 g O_2_ m^-2^ d^-1^; [9]) and Australian saline systems (4.4–17.5 g C m^-2^ d^-1^; [11]). However, oxygen production in Dziani Dzaha was less than the early estimates of [14] for the upper limits of photosynthetic productivity (43–57 g C m^-2^ d^-1^) in soda lakes. Lewis [16] proposed a value of 10 g C m^-2^ d^-1^ for maximum likely rate of photosynthesis for tropical and subtropical lakes, while limitations such as self-shading decrease the actual rates. In this study, carbon fixation was estimated to be close to this value, and, despite environmental limitations on photosynthesis (probable light penetration, nitrogen supply, carbon dioxide partial pressure for example) Dziani Dzaha primary production was close to the maximum attainable in this type of lake.

This high chlorophyll *a* biomass was dominated by a large, straight filamentous planktonic cyanobacterium belonging to the genus *Arthrospira* (up to 97.7% of the total biomass, Table 2). This genus is typically characterized by tightly or loosely coiled trichomes [39]. Transformations from helical to straight morphotypes have been observed in laboratory-grown cultures and production plants, however the control factors triggering such changes are still unknown [60, 61]. The genus *Arthrospira* is found in a wide range of saline habitats, and appears well adapted to saline-alkaline and even hypersaline environments [61]. *Arthrospira* is known to produce mass growth in many soda lakes in Africa as well as in alkaline lakes in Kenya [7]. This dominance affects the phytoplankton diversity, which is very low in Dziani Dzaha (up to 6 morphotypes were found during this survey). Extreme environments (e.g. high solar radiation, salinity, temperature and pH values) such as those found in Dziani Dzaha constitute selective pressures that probably allowed the dominance of this filamentous cyanobacterium. In such an environment, one should expect that they harbor specific metabolisms to help them withstand these extreme environments. Indeed, *Arthrospira* harbors particular metabolic pathways to withstand these harsh conditions [61, 62]. Characterizing the cyanobacterial metabolites in Dziani Dzaha communities or from isolated strains is one way of gaining a better understanding of their dominance and high biomass.

Despite high stocks and activities reported above, the renewal rate of photosynthetic biomass in Dziani Dzaha during the two surveys appeared to be relatively limited. Assuming a mean total particulate carbon of 35 mg C L^-1^ (Table 5) and a total photosynthetic carbon biomass of 140 g C m^-^^2^ for a 4 m deep water column, a daily production of 8.3 g C m^-^^2^ d^-1^ represented only 6% of the total stock. This allowed to an estimated population growth rate μ of 0.058 d^-1^, an extremely low value for cyanobacteria and phytoplankton as a general rule. At this growth rate and without biomass export, the cyanobacteria biomass would double every 13–14 days. If the hypothesis of a constant chlorophyll biomass is confirmed by further studies in Dziani Dzaha, the question of the fate of the organic matter produced remains to be solved, and heterotrophic bacteria, extremely abundant in the lake, would certainly play a critical role in carbon cycling despite relatively low biomasses compared to their densities (Table 7, Fig 4).

The abundance of heterotrophic bacteria appeared to be the same for both sampling dates and all depths, with an apparent diversity in shape and size (e.g. Fig 5D, epifluorescence microscopy with SybrGold staining and UV excitation). A mean abundance of 1.38 x 10^8^ cells mL^-1^ is much higher than in other aquatic ecosystems (estuaries, lakes and oceans), where values generally range from 1.4 10^5^ to 1.6 10^7^ cells mL^-1^ [63]. Flow cytometry did not clearly discriminated bacterial populations during the two surveys, except for the mean ratio of high to low DNA cells that differed in 2010 and 2011 by a factor of three. A high DNA content, revealed by the increase in fluorescence of SybrGreen® staining to nucleic acid, is commonly regarded as a consequence of active cell division. The underlying causes of such differences in our study are not known.

Apart from high cell counts in preserved samples, a significant number of diverse bacterial strains were isolated, cultivated and characterized using 16S rRNA gene sequencing. The diversity in cultivated strains from Dziani Dzaha is comparable to previous measurements in other saline lakes, including both halophiles and alkalinophiles such as *Halomonas*, *Marinobacter*, *Aliidiomarina* and *Nitrincola*, and species with wider distributions such as *Vibrio metschnikovii* or *Bacillus* spp. The cultivable eubacteria community is thus dominated by taxa usually found in saline and alkaline habitats, as expected for the properties of Dziani Dzaha water. Using culturing approaches coupled to 16S rRNA gene DGGE and sequencing, Dimitriu et al. [64] reported a similar dominance of Eubacteria by γ-proteobacteria in alkaline-saline Soap Lake, while a review by Jones et al. [65] highlighted that α-proteobacteria were generally absent from soda lakes. Culture-dependent identification of bacterioplankton is commonly regarded as highly selective but in the present study it allowed to bring out the uncommon Eubacteria community diversity in Dziani Dzaha.

The functional diversity potential of heterotrophic bacterioplankton, regarded as the utilization of carbon sources in Biolog Ecoplates, depended on the sampling year and depth. Based on ACWD values, anaerobic samples from deep water appeared to be generally the most efficient in metabolizing organic substrates. However, the high utilization rate of deep water samples was not the result of the utilization of a broad spectrum of carbon sources but was driven by the extremely high utilization of some compounds (here amino acids and carbohydrates) metabolized in anoxic conditions. The higher functional diversity was generally attributed to the physical, chemical and biological properties [50]. Easily degradable carbon sources were reported as mainly metabolized in the surface layers (where there are normally high DOC concentrations resulting from the primary production) while, in the deeper layers, microorganisms must utilize recalcitrant compounds such as organic polymers [66, 67]. In our study, the anoxic conditions at the bottom of Dziani Dzaha supported an active microbial community characterized by the preferential degradation of various polymers (tween 40, tween 80) and carbohydrates (mannitol, erythritol, and glycerol phosphate). It has already been shown that, as oxygen is depleted, microbial communities use carbohydrates in preference to carboxylic acids and amino acids [67]. Several other compounds such as glycogen and pyruvic acid were products or end-products of the anaerobic metabolism of glucose, here preferentially metabolized in samples AN10 and AN11. Little is known about the degradation of carboxylic acids in aquatic systems [68] but these compounds are considered part of the labile pool of organic matter [69], as confirmed by the present study. Of the 6 amino acids included in the microplates, glutamic acid, threonine and phenylalanine were efficiently degraded in the anoxic sample AN11, whereas asparagine and serine were the two preferred substrates for the oxic sample OX10. These differences in utilization efficiency can be explained by the high biodegradability of asparagine and serine through several metabolic pathways. In anoxic conditions, bacteria rely on oxidized electron acceptors such as NO_3_^-^ and SO_4_^2-^. Generally chemolithoautotrophic bacteria seem to adapt to these conditions but the incubation time in the Biolog plates was too short (72h) to allow the development of these microorganisms, because of their long generation time [70]. It is also clear that the high utilization of amino acids by the oxic samples (Table 6) was explained by the presence of nitrogen in these compounds, necessary for producing ammonium as a metabolite. In general, the ability of the heterotrophic microbial community to degrade low molecular weight substrates is consistent with the release of dissolved organic matter, both by exudation and cell lysis, as previously reported for example from cyanobacteria biomass [71].

The prokaryotic plankton of Dziani Dzaha is also characterized by a diverse archaeal community, with several Methanomicrobia, which originate mainly from anoxic environments (including mammal rumen, geothermal springs and activated sludge) where they produce methane. Unlike reported for most marine environments [72], no sequences affiliated to the Crenarchaeota phylum were found in the analyzed samples from Dziani Dzaha. This apparent dominance of Euryarchaeota, including extreme halophilic and methane producing Archaea, needs confirmation by implementing high throughput sequencing methods in further sampling surveys. In alkaline and saline environments Euryarchaeota have been reported to be the most abundant [65] and, unlike lakes with high sulfate content such as the temperate Soap Lake [64], the chemical nature of Dziani Dzaha waters does not seem to be compatible with high densities of sulfate-reducing bacteria that could outcompete methanogens. The thickness of the permanently anoxic layer in Dziani Dzaha allows the persistence of several anaerobic archaea, which are usually representative of anoxic benthic microflora [73].

## Conclusions

The physical and chemical characteristics of Dziani Dzaha are significantly different from other tropical soda and saline lakes, mainly owing to its extremely high alkalinity and the relative similarity of the dissolved element composition to seawater [74]. None of the standard criteria for extreme environments (temperature, salinity, pH) apply directly to this ecosystem but, in combination, the lake comes within the upper limits for inland aquatic ecosystems. Microbial life flourishes in this lake, and most of the carbon flow is driven by cyanobacterial photosynthetic activity. The primary production rates in Dziani Dzaha, calculated from the *in situ* dissolved oxygen evolution, are close to the upper limit predicted for inland waters [16] and appear to be limited by light availability and self-shading of biomass. In agreement with the diversity / productivity relationship for phytoplankton communities [75], the high biomass and production in Dziani Dzaha is associated with low phytoplankton diversity and the biomass is dominated by a single taxon represented by the filamentous cyanobacterium *Arthrospira*. In most tropical saline lakes, species belonging to the genus *Arthrospira* often appear coiled, and are responsible for high phytoplankton biomasses that are normally transferred to upper trophic levels [76–78]. A combination of polyphasic taxonomy methods using both ultrastructural characterization and 16S rRNA gene analyses is currently in progress to describe the members of the cyanobacteria community found in this lake. They will be used as a reference for analyzing the environmental genomic biodiversity of Dziani Dzaha in conjunction with spatial and temporal approaches.

The apparent stability of complex microbial communities is, in the present study, strongly supported by the positive feedback between high pH and high inorganic carbon availability for photosynthesis, as already described in alkaline systems dominated by cyanobacteria [8]. The high biomass and the quasi-extreme conditions act as a barrier for allochthonous microorganisms, possibly enhanced by the situation of the lake which is isolated on a small island with no river inlets. The local history of the ecosystem, including the geological context, has led to a self-regulating system, with several cyanobacteria, alpha- and beta-proteobacteria and Euryarchaeota phylogenetically unassigned. Metagenomic approaches are now required to estimate the microbial diversity, together with functional studies to evaluate the community turnover rate in Dziani Dzaha and identify the metabolic functions supported in the water column and superficial sediment. This will provide information about the nature (local *vs*. ubiquitous) of microbial components, and enable a comparison with other inland water bodies. Most of all, the definition of a core microbiome will also help to determine and understand the biogeochemical mechanisms related to the more global cycle that defined the original nature of Dziani Dzaha Lake waters. The findings reported here show that there is more to discover about the microbial diversity in this ecosystem, as, although the analysis methods and sampling were not extensive, several new taxa were found at various taxonomic levels, with known relatives from various habitats. These are the main objectives of a four year multi seasonal survey started in 2014 [74] to study microbial diversity, matter and energy flow in water column, surface gas exchanges, and sedimentary deposit in Dziani Dzaha.

## Supporting Information

S1 Text Isolation and identification of elements of the aerobic heterotrophic eubacterial community. (DOCX)

S2 Text Diversity of archaeal community in Dziani Dzaha. (DOCX)

S1 Table Chemical composition of saline lake waters selected for analysis. Fifty-two alkaline lakes samples (ascending alphabetical order) were retrieved from literature for this study.
(XLSX)

S1 Fig *In situ* dissolved oxygen records in Dziani Dzaha during a 48 h cycle in September 2011. Central panel: oxygen concentration evolution at 0.15 m (grey circles) and 0.5 m (black circles) depth in Dziani Dzaha Lake. Photosynthetically available radiation at 0.3 m is also given (X symbols). Upper panel: example of linearization of production periods, used to calculate photosynthetic oxygen production rates. Red lines indicate 95% confidence on production rate prediction. Lower panel: example of linearization of consumption periods, used to calculate oxygen respiration rates. Red lines indicate 95% confidence on respiration rate prediction.
(DOCX)
