# Supplementary material for: Microbial Diversity and Cyanobacterial Production in Dziani Dzaha Crater Lake, a Unique Tropical Thalassohaline Environment
Source: PLoS One. 2017 Jan 3;12(1):e0168879. doi: 10.1371/journal.pone.0168879 (PMC5207672; doi:10.1371/journal.pone.0168879)
Supplement: S1 Text — (DOCX) [file pone.0168879.s001.docx]

Leboulanger et al., Supplementary Information

**S1 Text.**

**Isolation and identification of elements of the aerobic heterotrophic eubacterial community**

For the cultivation of aerobic heterotrophic eubacteria, 1ml of the surface water was mixed with 10% glycerol and immediately put in liquid nitrogen. Back to the lab, 3 different solid media were designed. In order to mimic the chemical parameters of the lake, each medium was modified with a pH stabilized at 9.5 and salinity at 56 PSU. The 3 media used were: the Marine Agar (MA - Difco Laboratories, Detroit, USA), a Basalt Salt Methylotroph Medium (BSMM) and a mineral medium BG11 modified [1], [2] used for cyanobacteria isolation. In order to inhibit fungal growth, cycloheximide (100 mg/l) was added to each medium after autoclaving. The composition of the BSMM medium was prepared as follow (L^-1^): 50g of NaCl, 1.5g of MgCl_2_ x 6H_2_O, 20mg CaCl_2_ x 2H_2_O, 50mg NH_4_Cl, 100mg K_2_HPO_4_, 2g Na_2_SO_4_, 2g AMPSO and 15g of Agar. The pH was adjusted to 9.5 then, after autoclaving, 5ml/L of Methanol (HPLC grade -Sigma) as sole C source was added with also 1ml of a trace element mixture, vitamin mixture (K3129-SIGMA), Selenite-Tungstate solution and Lipoic acid solution. The trace element mixture was composed (L^-1^) of 12.5 ml HCl (25%), 2.1g FeSO_4_ x 7H_2_O, 144mg ZnSO_4_ x 7H_2_O, 100mg MnCl_2_ x 4H_2_O, 30mg H_3_BO_3_, 190mg CoCl_2_ x 6H_2_O, 2mg CuCl_2_ x 2H_2_O, 24mg NiCl_2_ x 6H_2_O, 36mg Na_2_MoO_4_ x 2H_2_O. The Selenite-Tungstate solution was prepared (L^-1^) with 0.4g of NaOH, 6mg of Na_2_SeO_3_ x 5H_2_O and 8mg of Na_2_WO_4_ x 2H_2_O. The lipoic acid solution was prepared with 15mg/L in a sodium phosphate buffer Na_2_HPO_4_ (10mM, pH7.1) and filter-sterilized (0.2µm).

100μl of the diluted (in sterile seawater) or undiluted sample was plated in triplicate on the 3 media and incubated in the dark at 25°C except the one plated on BG11 incubated under full light (40 μE m^−2^ s^−1^) during 2 weeks before counting and sub-culturing.

Identification by 16S rRNA gene sequencing

The identification of the most representative culturable strains was performed for each medium after 2 weeks. The colonies were categorized using morphologic characteristics. To ensure purification, all the different morphotypes (colony morphology) were picked for two successive sub-culturing steps, first onto the same medium and the last one on MA. Each isolate was then grown in marine broth (Marine Broth 2216, Difco Laboratories, Detroit, USA) for 48 h at 25 °C under agitation (100 rpm). Each culture was cryopreserved in 5 % dimethylsulfoxide or 35 % glycerol and put in −80 °C freezer and added to the MOLA culture collection (Microbial Observatory Laboratoire Arago, http://collection.obs-banyuls.fr/). For genomic DNA extraction, 2 ml of each liquid culture were spun down (10,000×g, 3 min). DNA extraction, PCR, and sequencing were done as previously described [3] using the BIO2MAR platform facilities (http://bio2mar.obs-banyuls.fr/en/index.html). Partial 16S rRNA gene sequences were trimmed manually, double checked, and dereplicated using the package Staden-GAP4 [4]. For bacterial strain identification, each FASTA file was uploaded in Ez Biocloud [5] and compared with the cultured bacterial strain database using Basic Local Alignment Search Tool (BLAST).

Partial sequences were aligned with MUSCLE in MEGA6 [6]. Then, phylogenetic relationship of all the sequences was determined using neighbour-joining [7]. The evolutionary distances were computed using the maximum composite likelihood method [8]. The resultant tree topologies were evaluated in bootstrap analyses of the neighbour joining method based on 1000 re-samplings [7]. Four different phylogenetic trees depending of the phylogenetic affiliation were constructed: Bacilli (Fig S1.1), Alphaproteobacteria (Fig S1.2), Gammaproteobacteria (Fig S1.3), and Bacteroidetes (Fig S1.4).

**Fig S1.1. Phylogenetic position of 16 strains distributed into the class *Bacilli.***

**Fig S1.2. Phylogenetic position of 11 strains distributed into the class *Alphaproteobacteria.***

**Fig S1.3.** **Phylogenetic position of 25 strains distributed into the class *Gammaproteobacteria.***

**Fig S1.4. Phylogenetic position of 6 strains distributed into the phylum *Bacteroidetes.***

**Table S1.1.** Accession numbers (GenBank) of 16S ribosomal RNA gene sequences of Eubacterial Strains (MOLA collection numbers: http://collection.obs-banyuls.fr/) isolated from Dziani Dzaha.

| Strain Number | Accession Number |  | Strain Number | Accession Number |  | Strain Number | Accession Number |
| --- | --- | --- | --- | --- | --- | --- | --- |
| MOLA1018 | KX818000 |  | MOLA1038 | KX818020 |  | MOLA1058 | KX818039 |
| MOLA1019 | KX818001 |  | MOLA1039 | KX818021 |  | MOLA1059 | KX818040 |
| MOLA1020 | KX818002 |  | MOLA1040 | KX818022 |  | MOLA1060 | KX818041 |
| MOLA1021 | KX818003 |  | MOLA1041 | KX818023 |  | MOLA1061 | KX818042 |
| MOLA1022 | KX818004 |  | MOLA1042 | KX818024 |  | MOLA1062 | KX818043 |
| MOLA1023 | KX818005 |  | MOLA1043 | KX818025 |  | MOLA1063 | KX818044 |
| MOLA1024 | KX818006 |  | MOLA1044 | KX818026 |  | MOLA1064 | KX818045 |
| MOLA1025 | KX818007 |  | MOLA1045 | KX818027 |  | MOLA1065 | KX818046 |
| MOLA1026 | KX818008 |  | MOLA1046 | KX818028 |  | MOLA1066 | KX818047 |
| MOLA1027 | KX818009 |  | MOLA1047 | KX818029 |  | MOLA1067 | KX818048 |
| MOLA1028 | KX818010 |  | MOLA1048 | KX818030 |  | MOLA1068 | KX818049 |
| MOLA1029 | KX818011 |  | MOLA1050 | KX818031 |  | MOLA1097 | KX818050 |
| MOLA1030 | KX818012 |  | MOLA1051 | KX818032 |  | MOLA1098 | KX818051 |
| MOLA1031 | KX818013 |  | MOLA1052 | KX818033 |  | MOLA1099 | KX818052 |
| MOLA1032 | KX818014 |  | MOLA1053 | KX818034 |  | MOLA1100 | KX818053 |
| MOLA1033 | KX818015 |  | MOLA1054 | KX818035 |  | MOLA1101 | KX818054 |
| MOLA1034 | KX818016 |  | MOLA1055 | KX818036 |  | MOLA1102 | KX818055 |
| MOLA1035 | KX818017 |  | MOLA1056 | KX818037 |  | MOLA1103 | KX818056 |
| MOLA1036 | KX818018 |  | MOLA1057 | KX818038 |  | MOLA1104 | KX818057 |
| MOLA1037 | KX818019 |  | MOLA1105 | KX818058 |  | ---- | ---- |

**References**

[1] Allen M.M. & Stanier R.Y. Growth and division of some unicellular blue-green algae. *J. Gen. Microbiol.* (1968) 51: 199-202

[2] Watanabe M.M., Kawachi M., Hiroki M. & Kasai F. *NIES-Collection List of Strains Sixth Edition 2000 Microalgae and Protozoa*. Microbial Culture Collections, National Institute for Environmental Studies, Tsukuba, (2000) 159 pp.

[3] Fagervold S. K., Urios L., Intertaglia L., Batailler N., Lebaron P. and Marcelino T. Suzuki. *Pleionea mediterranea* gen. nov., sp. nov., a gammaproteobacterium isolated from coastal seawater *Int J Syst Evol Microbiol* (2013) 63:2700-2705

[4] Staden R, Judge DP, Bonfield JK Managing sequencing projects in the GAP4 environment. In: Krawetz SA, Womble DD (eds) *Introduction to bioinformatics. A theoretical and practical approach*. Humana Press (2003), pp.

[5] Kim O.S., Cho Y.J., Lee K., Yoon S.H., Kim M., Na H., Park S.C., Jeon Y.S., Lee J.H., Yi H., Won S., Chun J.. Introducing EzTaxon: a prokaryotic 16S rRNA Gene sequence database with phylotypes that represent uncultured species. *Int J Syst Evol Microbiol* (2012) 62, 716–721

[6] Tamura K, Stecher G, Peterson D, Filipski A, and Kumar S. MEGA6: Molecular Evolutionary Genetics Analysis Version 6.0. *[Mol Biol Evol](http://mbe.oxfordjournals.org/content/30/12/2725.abstract.html?etoc)* (2013) 30: 2725-2729

[7] Felsenstein J. Confidence limits on phylogenies: an approach using the bootstrap. *Evolution* (1985) 39:783–791

[8] Tamura K, Nei M and Kumar S. Prospects for inferring very large phylogenies by using the neighbour-joining method. *Proc Natl Acad Sci* *(USA)* (2004) 101:11030–11035
