# Supplementary material for: Microbial Diversity and Cyanobacterial Production in Dziani Dzaha Crater Lake, a Unique Tropical Thalassohaline Environment
Source: PLoS One. 2017 Jan 3;12(1):e0168879. doi: 10.1371/journal.pone.0168879 (PMC5207672; doi:10.1371/journal.pone.0168879)
Supplement: S2 Text — (DOCX) [file pone.0168879.s002.docx]

Leboulanger et al., Supplementary Information

**S2 Text.**

**Diversity of archaeal community**

Prokaryotic genomic DNA from one sample (sampling date: 21/09/2011, water depth surface, station CLB) was extracted on 0.2 µm filter (20 mL prefiltred at 3.0 µm) using the Power Water DNA isolation kit (MoBio Laboratories) according to the manufacturer's instructions for maximum yields. DNA quality was checked by 1% (w/v) agarose gel electrophoresis and quantified using NanoDrop. The archaeal 16S rDNA full length genes were PCR-amplified using the archaeal specific forward primer Arch21F (5′-TCCGGTTGATCCYGCCGG-3′) and the universal reverse primer 1492R (5′-GGTTACCTTGTTACGACTT-3′) [1]. The PCR mix (50 µL) contained 1X PCR buffer, 2.5 mM MgCl_2_, 200 mM of each dNTP, 25 pmol of each primer, 250 ng mL^-1^ of bovine serum albumin (BSA, Sigma), 1.5 units of HotStart Taq DNA polymerase (Qiagen), and 10 ng of DNA extract. PCR reaction was carried out in a Labcycler SensoQuest. The thermal PCR profile was as follows: initial denaturation at 94°C for 15 min followed by 35 cycles of denaturation at 94°C for 1 min, primer annealing at 55°C for 1 min, and elongation at 72°C for 1 min. The final elongation step was 9 min at 72°C. The 16S rDNA products were analysed by electrophoresis in 1% (w/v) agarose gels. Then, the 16S rDNA product was purified (QIAquick PCR Purification Kit, Qiagen) before being cloned into a pGEM-T-Easy vector (Promega) to construct the clone library.

From the cloning step, 78 archaeal 16S rRNA gene forward sequences were obtained and clustered at the 97% similarity level using Mothur [2]. Twenty-six operational taxonomic units (OTU) at the 97% similarity level were selected and the 16S rRNA gene reverse sequences were sequenced from 3’ to 5’. The consensus sequences were constructed and we selected 17 representative clones at the 97% similarity cutoff using Mothur [2]. Selected sequences have been analysed using the ARB software package (version July 2014) [3] and the corresponding SILVA SSURef 99 database [4]. All sequences were automatically aligned using the SINA tool [5]. After importing aligned sequences, manual refinement of the alignment was carried out taking into account the secondary structure information of the rRNA. Aligned sequences were quickly added to the tree of the SILVA database. Tree reconstruction was first performed with 66 sequences > 1200 bp using three methods: neighbour joining (NJ, ARB), maximum parsimony (MP, PHYLIP DNA-Parsimony v 3.6a3; [6]) and maximum likelihood (ML, RAxML v8.0.24 [7] using CIPRES gateway [8]) in order to analyse the topology of the tree. The final tree was calculated with 46 sequences using a 50% archaeal positional conservatory filter + termini filter. Again, three methods were applied: ML (model: GTRGAMMA, bootstrapping: 1000 runs), NJ (distance: kimura, bootstrapping: 1000 replications) and MP (bootstrapping: 1000 runs) and topologies were compared. For better clarity, only selected subsets of the sequences used for treeing are shown in the Fig. S2.1.

All sequences obtained during this study are available from the databases of the INSDC, comprising DDBJ, EMBL and Genbank, under KT715014 to KT715030 accession numbers.


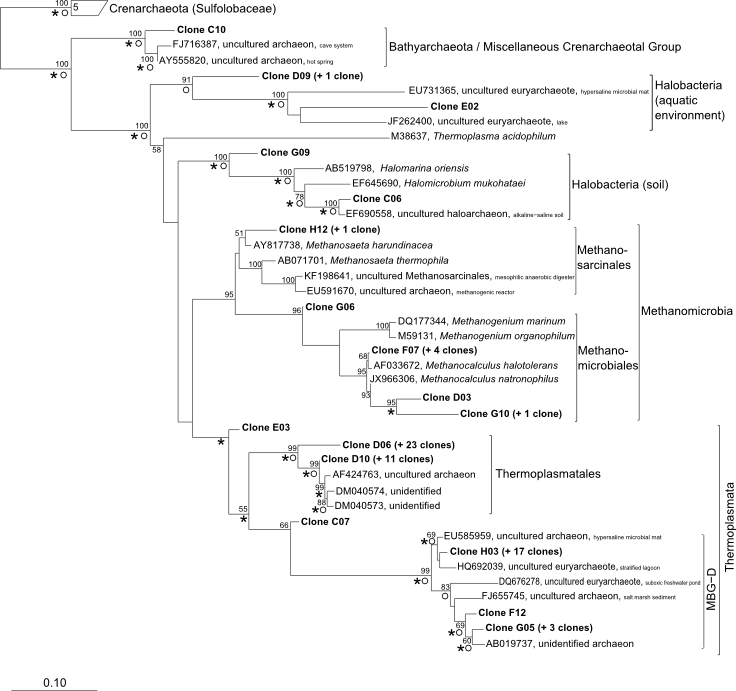


**Fig S2.1. Phylogenetic tree (maximum likelihood) constructed using 16S rDNA genes of Dziani Dzaha clones (in bold) and related species of Euryarcheaota and Bathyarcheaota group (provided with accession number).**

Bar indicated 10% of dissimilarity sequence. Selected sequences from the Crenarchaeota group were used to root the tree. Bootstrap percentages are obtained using 1000 replicates, and values >50% are indicated at the nodes, * and indicated branches that were also found using the maximum parsimony and the neighbour-joining method, respectively.

**References**

[1] Delong EF. Archaea in coastal marine environments. Proc Natl Acad Sci USA. 1992; 89: 5685-89

[2] Schloss PD, Westcott SL, Ryabin T, Hall JR, Hollister EB et al. Introducing mother: open-source, platform-independent, community-supported software for describing and comparing microbial communities. Appl Environ Microbiol. 2009; 75: 7537-41

[3] Ludwig W, Strunk O, Westram R, Richter L, Meier H, Yadhukumar et al. ARB: a software environment for sequence data. Nucleic Acid Res*.* 2004; 32: 1363-71

[4] Yilmaz P, Parfrey LW, Yarza P, Gerken J, Pruesse E, Quast C et al. The SILVA and “All-species Living Tree Project (LTP)” taxonomic frameworks. Nucleic Acid Res. 2014; 42: D643-648

[5] Pruesse E, peplies J, Glöckner FO. SINA: accurate high-throughput multiple sequence alignment of ribosomal RNA genes. Bioinformatics. 2012; 28: 1823-29

[6] Felsentein F. Inferring phylogenies. Sunderland, Mass. Sinauer Associate Inc. publishers. 2004.

[7] Stamatakis A. RAxML version 8: a tool for phylogenetic analysis and post-analysis of large phylogenies. Bioinformatics. 2014; 30: 1312-13

[8] Miller MA, Pfeiffer W, Schwartz T. Creating the CIPRES science gateway for inference of large phylogenetic trees. In: Gateway Computing Environments (GCE) Workshop*.* 2010; pp. 1-8
