# Supplementary material for: Microbial Diversity and Cyanobacterial Production in Dziani Dzaha Crater Lake, a Unique Tropical Thalassohaline Environment
Source: PLoS One. 2017 Jan 3;12(1):e0168879. doi: 10.1371/journal.pone.0168879 (PMC5207672; doi:10.1371/journal.pone.0168879)
Supplement: S1 Fig — Central panel: oxygen concentration evolution at 0.15 m (grey circles) and 0.5 m (black circles) depth in Dziani Dzaha Lake. Photosynthetically available radiation at 0.3 m is also given (X symbols). Upper panel: example of linearization of production periods, used to calculate photosynthetic oxygen production rates. Red lines indicate 95% confidence on production rate prediction. Lower panel: example of linearization of consumption periods, used to calculate oxygen respiration rates. Red lines indicate 95% confidence on respiration rate prediction. (DOCX) [file pone.0168879.s004.docx]

Leboulanger et al., Supplementary Information

**S1 Fig.**

**S1 Fig. *In situ* dissolved oxygen records in Dziani Dzaha during a 48 h cycle in September 2011.** Central panel: oxygen concentration evolution at 0.15 m (grey circles) and 0.5 m (black circles) depth in Dziani Dzaha Lake. Photosynthetically available radiation at 0.3 m is also given (X symbols). Upper panel: example of linearization of production periods, used to calculate photosynthetic oxygen production rates. Red lines indicate 95% confidence on production rate prediction. Lower panel: example of linearization of consumption periods, used to calculate oxygen respiration rates. Red lines indicate 95% confidence on respiration rate prediction.
